# An On‐Chip Quad‐Wavelength Pyroelectric Sensor for Spectroscopic Infrared Sensing

**DOI:** 10.1002/advs.201900579

**Published:** 2019-08-26

**Authors:** Thang Duy Dao, Satoshi Ishii, Anh Tung Doan, Yoshiki Wada, Akihiko Ohi, Toshihide Nabatame, Tadaaki Nagao

**Affiliations:** ^1^ International Center for Materials Nanoarchitectonics (MANA) National Institute for Materials Science (NIMS) 1‐1 Namiki, Tsukuba Ibaraki 305‐0044 Japan; ^2^ Department of Condensed Matter Physics Graduate School of Science Hokkaido University Kita 10, Nisi 8, Kita‐ku Sapporo 060‐0810 Japan; ^3^ Research Center for Functional Materials National Institute for Materials Science (NIMS) 1‐1 Namiki, Tsukuba Ibaraki 305‐0044 Japan; ^4^ Nanotechnology Innovation Station National Institute for Materials Science (NIMS) 1‐1 Namiki, Tsukuba Ibaraki 305‐0044 Japan

**Keywords:** on‐chip sensors, perfect absorbers, plasmonic metamaterials, pyroelectric sensors, wavelength‐selective infrared sensors

## Abstract

Merging photonic structures and optoelectronic sensors into a single chip may yield a sensor‐on‐chip spectroscopic device that can measure the spectrum of matter. In this work, an on‐chip concurrent multiwavelength infrared (IR) sensor, which consists of a set of narrowband wavelength‐selective plasmonic perfect absorbers combined with pyroelectric sensors, where the response of each pyroelectric sensor is boosted only at the resonance of the nanostructured absorber, is proposed and realized. The proposed absorber, which is based on Wood's anomaly absorption from a 2D plasmonic square lattice, shows a narrowband polarization‐independent resonance (quality factor – *Q* of 73) with a nearly perfect absorptivity as high as 0.99 at normal incidence. The fabricated quad‐wavelength IR sensors exhibit four different narrowband spectral responses at normal incidence following the predesigned resonances in the mid‐wavelength infrared region that corresponds to the atmospheric window. The device can be applied for practical spectroscopic applications such as nondispersive IR sensors, IR chemical imaging devices, pyrometers, and spectroscopic thermography imaging.

## Graphical abstract

**An approach to realize on‐chip multiwavelength infrared (IR) sensors** is reported. As a proof of concept, the fabricated quad‐wavelength IR sensors exhibit four different narrowband spectral responses at normal incidence. The multiwavelength IR sensors can be applied for portable spectroscopic devices such as nondispersive IR sensors, IR chemical imaging, pyrometers, and thermography cameras.

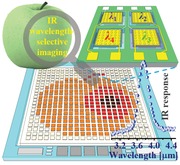

## 1 Introduction

Infrared (IR) spectroscopy is one of the most powerful spectroscopic techniques to identify chemical species by analyzing their vibrational absorptions, as well as to characterize optical properties of narrow bandgap semiconductors, quantum well emitters, and thermal absorbers. The performance of an IR spectrometer, which includes the resolution, sensitivity, and measurement time, relies on the massive spectroscopic modules such as prisms, diffraction gratings, interferometers, mechanical scanners, and goniometers, as well as on their detection modules. Recent advances in photonics and nanofabrication techniques can lead to other types of IR spectroscopic devices in which their dispersive elements and IR detectors are integrated into a compact microdevice for portable yet accurate spectroscopic applications; such as nondispersive infrared (NDIR) sensors, dual‐wavelength pyrometers, as well as chemical IR imaging integrated with thermography devices. The most important requirement of such devices is the dispersive element working at normal incidence, which must be simple, and has a narrow bandwidth with a clean background in a broad spectral range. Thence, the interference filter is one of the most common choices. However, adding such macroscopic interference filters before IR sensors limits the number of the resonant wavelength for precise and multifunctional IR spectroscopic devices. Another approach that has been advanced as a possible solution to the requirement of this handheld concurrent multiwavelength IR sensor, is to employ novel photonic crystals^1^, ^2^, ^3^, ^4^ and plasmonic structures,^5^, ^6^, ^7^, ^8^, ^9^, ^10^ wherein the field confinement and resonant bandwidth as well as the resonant tunability can be controlled more easily in micrometer‐scale devices. Nevertheless, the most photonic and plasmonic structures are rather complex structures and have been applied only for individual single‐wavelength IR sensors. Moreover, their bandwidths are far wider than those of conventional spectrometers that possess resolutions narrower than the widths of molecular vibrations. These are the major impediments for realizing miniature sensor‐on‐chip spectroscopic devices, and thus, simple small‐size spectroscopic modules with ultranarrowband detection and broad tunability in the IR spectral range is strongly required.

In recent years, resonant plasmonic metamaterial absorbers have attracted a great interest in the field of photonics due to their versatile ability in achieving near‐unity absorption and in controlling the resonant bandwidth as well as tunability.^11^ They have shown impressive practical applications involving solar energy harvesting,^12^, ^13^, ^14^ thermal photovoltaics,^15^, ^16^, ^17^ thermal emission,^18^, ^19^, ^20^, ^21^ radiative cooling,^22^, ^23^, ^24^ NDIR spectroscopy,^25^, ^26^ amplifying signals in IR spectroscopy,^27^, ^28^ as well as IR sensors.^29^, ^30^, ^31^, ^32^, ^33^, ^34^ Particularly, when an electromagnetic field is absorbed by a perfect absorber, the absorbed energy is eventually converted into heat through Joule heating that follows Poynting's theorem.^35^, ^36^ With a nonmagnetic absorptive dielectric resonant absorber, the dissipative energy density is proportional to the resonant angular frequency – ω and the imaginary part – Im ε(ω) of the dielectric function of the medium^37^, ^38^, ^39^

(1) Q = 12ε0ωIm εωE→ 2

where ε_0_ is the electric constant in vacuum. Thus, a perfect absorber at the resonance can be an efficient light‐to‐heat transducer. By integrating individual single‐wavelength resonant perfect absorbers with thermal detectors into a single chip, the multiwavelength IR devices can be feasible.

In this work, we first discuss the possible absorber structures that are compatible for on‐chip multiwavelength thermal sensors. Then, we propose a conceptual design of sensor‐on‐chip infrared spectroscopic devices utilizing Wood's anomaly absorption from 2D periodic metallic arrays, which are directly mounted on individual pyroelectric ZnO transducer for efficient light‐to‐heat conversion and efficient heat transfer. The 2D plasmonic absorber exhibits an ultranarrowband polarization‐independent resonance at the normal incidence in the mid‐wavelength infrared (MWIR) region with a high *Q*‐factor of 73, and with a nearly perfect absorptivity as high as 0.99. As a proof of concept of the spectroscopic microdevices for next generation MWIR sensors, we fabricated a set of on‐chip, membrane‐supported quad‐wavelength infrared sensors on a 3 in. Si wafer. The fabricated quad‐wavelength infrared sensors exhibited sharp spectral responses at the wavelengths exactly matching to the resonance wavelengths at the normal incidence. We also provide a detailed fabrication procedure of the device, which can also be applicable for scaling down the device to sub‐hundred micrometer scale, making the proposed devices suitable for miniature spectroscopic sensors.

## 2 Design Strategy of Ultranarrowband Perfect Absorbers Aiming for Wavelength‐Selective Thermal Sensors

**Figure 1:**
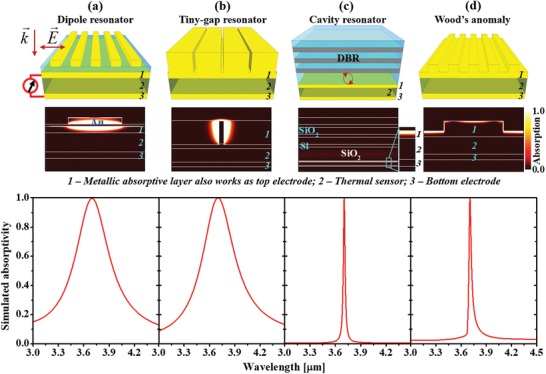
Comparison of four different perfect absorbers for IR wavelength‐selective thermal sensors which have resonances at around 3.7 µm. From top to bottom: device structures, simulated absorption maps, and absorptivity spectra of the four absorbers. a) Metal dipole antenna–insulator–metal absorber. b) Tiny‐gap plasmonic resonator array exhibiting unity absorption peak with *Q‐*factors of about 10. c) Asymmetric cavity absorber with the top reflector made of a distributed Bragg reflector (DBR). d) Wood's anomaly (grating‐coupled SPP) exhibiting much narrower unity absorption peak with *Q*‐factor as high as ≈150.

In the beginning, we examine four different perfect absorber configurations that are compatible with thermal sensors for sensor‐on‐chip MWIR spectroscopic devices. The requirements to achieve a wavelength‐selective absorber are the confined resonator and the intrinsic loss of the medium. In this concern, resonators consisted of a lossless dielectric and a low‐loss metal are the most common structures for perfect absorbers.^11^, ^18^, ^19^, ^20^ **Figure** **1** indicates four different perfect absorbers that can be combined with thermal sensors such as pyroelectric, bolometric, or thermoelectric materials for on‐chip IR spectroscopic devices. As seen in Figure 1a, a metal–insulator–metal absorber with a plasmonic dipole resonator array on the top shows a perfect resonant absorption peak with a *Q*‐factor of 10. In this configuration, the radiation is absorbed in both the top resonant antenna and the bottom metal mirror. Due to the low‐thermal conductivity of the insulator, the heat induced at the top resonant antenna may not efficiently transfer to the thermal sensor. Figure 1b shows another perfect absorber configuration utilizing the nearfield confinement in a tiny‐gap array that exhibits the similar *Q*‐factor and absorptivity. This design can improve the conductive heat transfer from the absorber to the thermal sensor. However, this structure requires the gaps to be a few tens of nanometers in width and 500 nm in height, which is quite challenging to fabricate. It should be noted that, the resonant bandwidths of these absorbers (Figure 1a,b) are much broader compared to that of typical molecular vibrations, which severely limits the application of these spectroscopic sensors.

By contrast, absorbers incorporating periodic structures can significantly narrow the resonance bandwidth. For example, Figure 1c,d shows two different periodic structures that exhibit ultranarrowband resonance with *Q*‐factors as high as 150, which are comparable to the most molecular vibrations in solid state. Since the sharp resonance of the cavity absorber shown in Figure 1c is embodied by the layered photonic structure, it is not straightforward to fabricate cavities having different resonance wavelengths on a single chip. Keeping this limitation in mind, in Figure 1d we consider Wood's anomaly absorption in a 1D metallic grating structure which is realized by the surface‐wave resonance condition between the surface plasmon polariton (SPP) and 1D grating.^40^, ^41^, ^42^, ^43^, ^44^ The heat induced in the metallic array directly transfers to the thermal sensor module without being blocked by low thermal‐conductivity object. The perfect absorption is realized by designing the depth and shape of the unit cell structure, and its resonance wavelength and directivity can be flexibly tuned by its periodicity. These features make the Wood's anomaly perfect absorber advantageous compared to the other three absorbers in the on‐chip multiwavelength IR sensor application. It should be noted that the three 1D periodic absorbers shown in Figure 1a,b,d are polarization‐dependent, yet polarization‐independent absorbers can be achieved by extending each design into 2D periodic array. Therefore, a 2D periodic plasmonic array can be a structure of choice for designing sensor‐on‐chip spectroscopic devices with polarization‐independent ultranarrowband absorption.

## 3 Results and Discussions

### 3.1 The Design and Simulation of the Quad‐Wavelength IR Sensor

**Figure 2:**
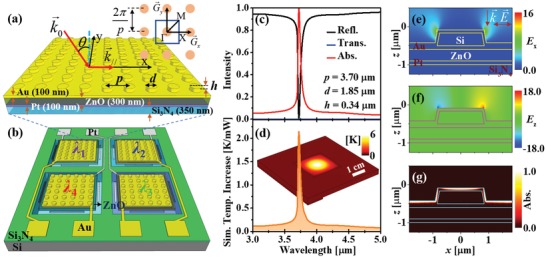
a) Schematic illustration of the WAA‐IR sensor. b) Schematic design of the on‐chip quad‐wavelength membrane pyroelectric sensor. c) Simulated reflectance, transmittance, and absorptivity of a 3.7 µm periodicity absorber indicate a narrow and perfect absorption. d) Simulated heat generation spectrum averaged in the ZnO pyroelectric layer of a 3.7 µm periodicity sensor excited by a 0.5 mm radius gaussian beam with a power of 1 mW. The inset in (d) reveals a 3D heat increase distribution of the sensor. e–g) Simulated distributions of electric fields (*E_x_*, *E_z_*) and absorption excited at the resonance (3.722 µm) of a 2D plasmonic array absorber.

The conceptual design of the Wood's anomaly absorber (WAA) quad‐wavelength IR sensor is illustrated in **Figure** **2**a,b. Here, a set of four 2 × 2 mm^2^ wavelength‐selective pyroelectric sensors with different resonances are arranged into a 1 × 1 cm^2^ silicon‐based substrate (Figure 2b). Depending on the application, the size of each sensor chip can be scaled down to sub‐hundred micrometers. The thermal insulation of each resonant sensor is achieved by a Si_3_N_4_ membrane (350 nm thick) with four slits (20 µm width) on the edges of each sensor. In each sensor, a 2D periodic plasmonic disk‐on‐film array made of gold (Au) works as a narrowband perfect absorber to absorb IR radiation at a resonant wavelength designed under the normal incidence. The absorbed electromagnetic radiation is converted into heat through Joule heating (resistive heat) following the Poynting's theorem described earlier. Hence, the resistive heat in the resonant metal absorber conductively transfers to the ZnO pyroelectric sensor (300 nm thick) to boost electrically polarized charges in the pyroelectric film, generating a temporary voltage at the both ends of the ZnO layer. The temporary voltage is then measured through the top (Au) and bottom (Pt) contacts (100 nm thick for each film) as the IR sensing signal. The cooling process also gives rise to a reversed temporary voltage signal. The resonance of each sensor relies on the diameter, *d*, the height, *h*, and the periodicity, *p*, of the metallic disk‐on‐film array.

The optical resonance of the WAA can be explained through the SPP‐photonic coupling in a 2D periodic plasmonic lattice. In a plasmonic square lattice (see the inset in Figure 2a for the reciprocal space), SPPs at the metal–air interface are given by

(2) $$\left|{\vec{k}}_{\mathrm{spp}}\right|\;=\;k_{0}\sqrt{\frac{\varepsilon_{m}}{\varepsilon_{m}+1}}$$

where ε_m_ is the complex permittivity of metal and $k_{0}\;=\;\frac{2\pi }{\lambda }$. SPPs are excited if their momentums match the momentum of the incident photon and the periodic lattice (momentum conservation)

(3) $${\vec{k}}_{\mathrm{spp}}\;=\;{\vec{k}}_{∥}+i{\vec{G}}_{x}+j{\vec{G}}_{y}$$

where $|{\vec{k}}_{∥}|\;=\;k_{0}\sin\theta$ is the projection of momentum of the excitation photon with an incident angle of θ on the metal surface, ${\vec{G}}_{x}$ and ${\vec{G}}_{y}$ are the two primitive lattice vectors, *i* and *j* are integers. In case ${\vec{k}}_{∥}$ is oriented along ${\vec{G}}_{x}$ (Γ−X direction, see the inset in Figure 2a), Equation (3) can be written as

(4) $${\left|{\vec{k}}_{\mathrm{spp}}\right|}^{2}\;=\;{\left|\left({\vec{k}}_{∥}+i{\vec{G}}_{x}\right)+j{\vec{G}}_{y}\right|}^{2}\;=\;{\left(k_{0}\sin\theta +iG_{x}\right)}^{2}+{\left(jG_{y}\right)}^{2}$$

From Equations (2) and (4), and with $|\vec{G_{x}}|\;=\;|\vec{G_{y}}|\;=\;\frac{2\pi }{p}$, $k_{0}\;=\;\frac{2\pi }{\lambda }$, the angular dependent dispersion relation of SPPs for a square lattice along Γ−X direction is expressed by

(5) $$\frac{\varepsilon_{m}}{\varepsilon_{m}+1}\;=\;\sin^{2}\theta +\left(\frac{2i}{p}\sin\theta \right)\lambda +\left(\frac{i^{2}+j^{2}}{p^{2}}\right)\lambda^{2}$$

At normal incidence, the resonant wavelength is simply calculated by $\lambda \;=\;\frac{p}{\sqrt{i^{2}+j^{2}}}\sqrt{\frac{\varepsilon_{m}}{\varepsilon_{m}+1}}$. Therefore, at normal incidence, the resonant wavelength of the WAA IR sensor is simply predicted by the periodicity – *p*, although the height and disk size affect the resonance strength. Because of the coupling nature of the plasmonic surface waves with the periodic property, the resonance in the disk‐on‐film 2D WAA is much narrower compared to the localized plasmon resonance in other absorber structures as proven in Figure 1a,b. From the numerical simulations based on the rigorous coupled‐wave analysis (RCWA), we verified that the bandwidth and the absorptivity of the 2D WAA mainly depend on the diameter – *d* and the height – *h* of the metallic disk as shown in Figure S1a,b (Supporting Information), respectively. In this quad‐wavelength IR sensor, we kept the metallic disks of both four absorbers at the same height and the diameter at a half of the periodicity. Figure 2c shows the simulated optical spectra of a disk‐on‐film 2D WWA IR sensor having a periodicity of 3.7 µm, a disk diameter of 1.85 µm and a disk height of 0.34 µm under the normal incident excitation. It is worth noting that we adopted a thin Au‐covered Si disk array instead of using a sub‐micrometer‐thick Au disk array, which exhibits the same optical properties while saving more Au compared to the identical disk array only made of Au (Figure S2, Supporting Information). As seen from the figure, the plasmonic absorber with 3.7 µm periodicity exhibits a nearly perfect absorptivity (0.99) resonant peak at 3.722 µm with a narrow bandwidth of 51 nm (*Q*‐factor of 73). With a symmetrical geometry of the 2D plasmonic square lattice adopted here, the WAA does not depend on the polarization (Figure S3a, Supporting Information), which makes the wavelength‐selective sensor more applicable in the multifunctional practical applications. The simulated angle‐dependent absorptivity of the WAA shown in Figure S3b (Supporting Information) indicates clearly the hybridized nature of SPP waves with the photonic property in a 2D plasmonic square lattice as predicted in Equation (5) (white‐dashed curves in Figure S3b, Supporting Information).

In order to further elucidate the SPP‐photonic coupling in the 2D WAA, electric field distributions in the plasmonic structure were calculated using a full‐wave simulation based on the finite‐difference time‐domain (FDTD) method. Figure 2e,f shows the simulated electric field distributions (*E_x_* and *E_z_*) of a 3.722 µm resonant sensor excited at the resonance under normal incidence. It is clearly seen that strong electric nearfields are converged at the edges of the metallic disks. Furthermore, the induced electric field – *E*_z_ (Figure 2f) resulted from the coupling between the incident photon and the 2D periodic metallic lattice reveals strongly coupled nearfields with their phases alternating along the *x*‐axis, evidencing that the SPP waves are excited and propagated along the metallic surface. The SPP waves are then strongly damped via metallic losses (Figure 2g) at the Au surface, resulting in a narrow absorption as shown in Figure 2c. This resistive absorption in the metal generates heat due to Joule heating, then heat is conductively transferred to the pyroelectric ZnO layer, producing the temporary voltage signal. The combination of highly selective excitation of grating‐coupled surface waves and its swift plasmonic damping is the effective mechanism of the perfect absorption with very high wavelength selectivity. To understand the light‐heat conversion and the heat transfer processes in the sensor, we performed multiphysics simulations for the periodic array absorber sensor membrane. Figure 2d and its inset, respectively, present the simulated spectrum of the heat increase averaged in the pyroelectric ZnO layer and the heat distribution of a 3.722 µm resonant sensor irradiated at the resonance by a 0.5 mm radius gaussian beam with a power of 1 mW. Interestingly, the averaged heat spectrum in the ZnO sensor reveals a resonance at 3.722 µm, which is exactly the same as the simulated absorption spectrum (Figure 2c). Furthermore, the averaged heat induced by the 2D WAA at the pyroelectric layer increases as high as 2.3 K at thermal equilibrium when it is irradiated at the resonance with a power of 1 mW, which is certainly high enough for the thermal sensor response. Thus, the conceptual design of the quad‐wavelength IR sensor is proved and can be adopted for practical applications.

### 3.2 Fabrication of MEMS‐Based Quad‐Wavelength IR Sensor

**Figure 3:**
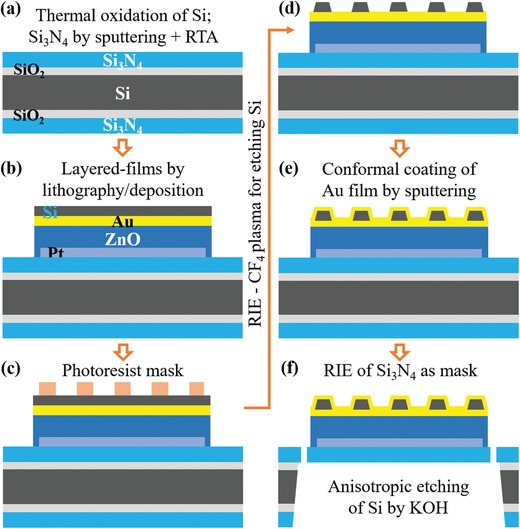
Schematic fabrication process of the membrane WAA‐IR sensor. a) Double side polished 3 in. Si wafer with a 100 nm thermally oxidized layer and a 350 nm sputtered Si_3_N_4_ film on both sides. b) Patterning bottom Pt electrode, ZnO pyroelectric film, top Au electrode, and Si template film using direct laser writing lithography combined with film deposition and lift‐off processes. c) Patterning photoresist disk array as RIE mask. d) Patterning Si disk array by RIE with photoresist mask array. e) Conformal coating of Au layer on Si disk array using sputtering. f) RIE of top Si_3_N_4_ mask for thermal isolation slits, and bottom Si_3_N_4_ mask for anisotropic etching of Si, then KOH anisotropic etching of Si for membrane.

To realize the proposed quad‐wavelength IR sensor, several steps of direct laser writing lithography involving the film deposition and lift‐off, the reactive‐ion etching (RIE), and the anisotropic wet‐etching were carried out on a 3 in. double side polished Si substrate, where a set of 25 quad‐wavelength IR sensors were arranged. The fabrication procedure is depicted in **Figure** **3**, and the details are given in the Experimental Section. As discussed above, instead of using a tall Au disk (340 nm) array for the 2D WAA, an 80 nm thick Au film coated on a 340 nm thick Si disks array was adopted to save gold while it retains essentially identical performance to the tall Au disk array. The periodicities of four plasmonic array sensors with their resonances aiming at the transparent atmospheric window in the MWIR region are 3.5, 3.7, 3.8, and 3.9 µm. Here we use a 100 nm thick Pt film deposited by electron beam evaporation as the bottom electrode, wherein the film also works as an epitaxial substrate with (111) face for growing highly crystalline ZnO(0001) film.^31^, ^45^

**Figure 4:**
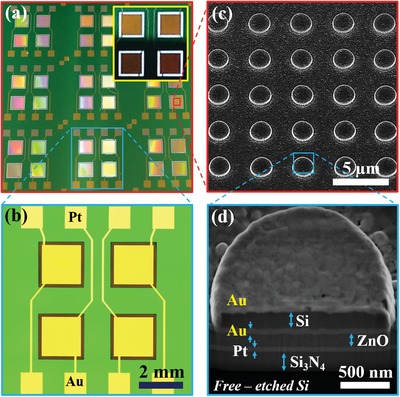
a) A photo of nine fabricated quad‐wavelength membrane pyroelectric sensors. The inset presents a photo of a quad‐wavelength membrane sensor where clear transparent white light through the Si_3_N_4_ membrane is visible. b) Optical microscopy image of a quad‐wavelength membrane sensor. c) Top‐view SEM image of the fabricated 3.7 µm periodic resonance sensor. d) Cross‐sectional tilted‐view SEM image of a membrane sensor.

**Figure** **4** summarizes the morphological characterizations of the fabricated quad‐wavelength IR membrane sensors. Figure 4a is a photo viewed at a set of nine fabricated quad‐wavelength IR sensor chips from a whole 3 in. wafer. The inset in Figure 4a shows a photo of a typical fabricated quad‐wavelength sensor taken under the white light illuminated from the bottom, which reveals the optically transparent Si_3_N_4_ membrane layer around each single‐wavelength sensor, indicating the fabricated sensors were well suspended from the Si substrate by the Si_3_N_4_ membrane. Figure 4b shows a bright‐field optical microscope image scanned over a sensor chip with a scale bar of 2 mm, which approves the dimensional parameters of the fabricated sensor of 2 × 2 mm^2^ for each single‐wavelength sensor and of 1 × 1 cm^2^ for a whole quad‐wavelength sensor. A top‐view scanning electron microscopy (SEM) image of a sensor presented in Figure 4c displays a typical fabricated WAA‐IR sensor. A cross‐sectional view shown in Figure 4d explores the structural view of the sensor, which clearly evidences that the Au‐shell disk supported by the Si‐core template was well‐constructed using the proposed fabrication process described above. The dimensional parameters of each film layer in the sensor, including the plasmonic disk array, the Au top electrode, the pyroelectric ZnO film, the bottom Pt electrode as well as the Si_3_N_4_ membrane are also clearly seen and verified in Figure 4d, which are the same as designed.

### 3.3 Proof of Concept

**Figure 5:**
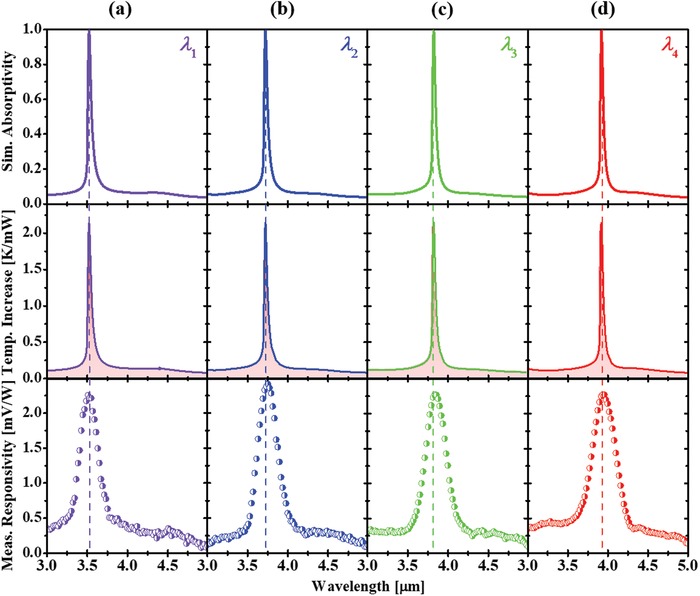
a–d) Optical and electrical response of a quad‐wavelength membrane pyroelectric sensor with resonances at: a) 3.522 µm (λ_1_), b) 3.722 µm (λ_2_), c) 3.822 µm (λ_3_), and d) 3.922 µm (λ_4_). From top to bottom panels: simulated typical absorptivity spectra; simulated heat increase spectra averaged in ZnO layer at thermal equilibrium; and measured spectral response curves of both four wavelengths.

The performance (spectral response) of the fabricated quad‐wavelength sensors was characterized using a tunable IR laser system. The measurement setup is detailed in the Experimental Section. **Figure** **5**, from top to bottom, presents the simulated absorptivity spectra, the simulated averaged heat increase spectra and the measured spectral response curves, respectively, of four individual single‐wavelength sensors of a typical quad‐wavelength sensor. Four single‐wavelength sensors were labeled as follows depending on the resonance wavelengths; 3.522 µm (λ_1_, Figure 5a), 3.722 µm (λ_2_, Figure 5b), 3.822 µm (λ_3_, Figure 5c), and 3.922 µm (λ_4_, Figure 5d). Here we used the same disk height, *h*, of 340 nm for all the four resonant absorbers, but with different periodicities, *p*, of 3.5 µm (λ_1_), 3.7 µm (λ_2_), 3.8 µm (λ_3_), and 3.9 µm (λ_4_) wherein each disk diameter was fixed at a half of each periodicity. As seen in Figure 5 (top panels), the simulated responsivity spectra of the quad‐wavelength sensor are almost unity (0.99) and narrow (50 nm), which proves that the designed sensor can efficiently absorb IR light at each resonance. Indeed, the simulated temperature increase averaged over the pyroelectric ZnO film at thermal equilibrium taken from both four individual single‐wavelength sensors (middle panels in Figure 5) clearly shows that the quad‐wavelength sensor can efficiently absorb IR light in the narrow spectral bandwidth at the designed resonances, then converts the absorbed resonant IR energy into heat with respect to the absorption spectra, and the induced heat subsequently transfers to the ZnO sensing layer. As expected, the measured spectral response curves shown in the bottom panels of Figure 5 clearly prove the conceptual design of the on‐chip multiwavelength sensor proposed in this work. The four‐individual single‐wavelength sensors in a quad‐wavelength sensor chip exhibit narrow responsivity curves in which their resonances agree well with the simulated absorptivity spectra as predesigned. The broadening of the measured spectral response curves compared to the simulated absorptivity and temperature increase spectra is due to the broad spectral linewidth (*Q*‐factor of 10–15) of the IR laser used in the measurement.

**Figure 6:**
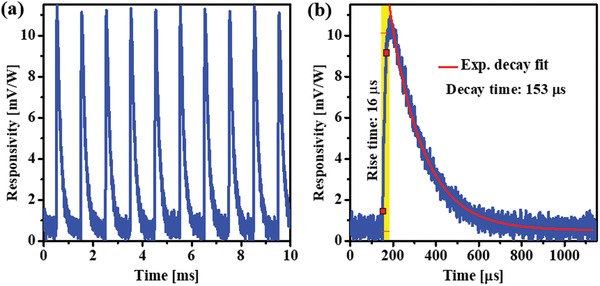
Measured temporal response of a 3.722 µm resonant sensor chip excited by a 104 fs pulsed laser at resonance. a) The stability of the sensor response within ten pulses duration. b) The impulse response of the sensor at a single pulse reveals a rise time of 16 µs and a decay time of 153 µs.

Since the resonance of the spectroscopic sensor chip was originated from the Wood's anomaly absorption in the 2D periodic plasmonic array, which is angle‐dependent, we further performed the angle‐dependent responsivity measurement to verify the angular response characteristic of the sensor. Figure S4 (Supporting Information) plots the spectral response curves of a 3.7 µm periodicity wavelength‐selective sensor measured at normal incidence and at 5° and 10° tilted angles. Interestingly, despite the angle‐dependent resonance, the responsivity of the sensor at oblique incidence decreases significantly when the incident angle increases, indicating that the fabricated wavelength‐selective sensor has high directivity at normal incidence. To understand the dynamic response of the device, the temporal response of a 3.722 µm resonant sensor was experimentally measured using a high‐performance oscilloscope. As seen in **Figure** **6**, the uniform response of the sensor measured within ten pulses (Figure 6a) indicates the fast response and the stability of the sensor. The impulse response of the sensor measured with single pulse excitation (Figure 6b) shows a fast response time of 16 µs and a decay time of 153 µs, which are applicable to the practical devices.

### 3.4 Potential Applications

With the accessible design and narrowband responsivity at resonances, the sensor‐on‐chip quad‐wavelength IR device developed in this work can be extend into multiwavelength sensors for practical applications in portable IR spectroscopic devices such as in the true‐temperature pyrometry,^46^ IR color imaging for environmental detection and materials recognitions,^47^ as well as IR remote sensing of atmospheric pollutions for controlling air quality.^48^, ^49^, ^50^ Although the current 2D WAA‐based multiwavelength sensor already shows high directivity at normal incidence, the performance can be further improved by adopting a pinhole aperture, or collimator. Furthermore, if the device is operated with a monochromatic light at its resonance wavelength, it shows an ultranarrow working angle (below 1°, see Figure S5, Supporting Information), in which one may find angular‐sensitive sensing applications such as angular positioning or automotive devices. The current sensor size is on the order of millimeter, however, the sensor can be scaled down to sub‐hundred micrometer with the identical design and fabrication, and the ZnO layer can be replaced by other thermal sensors such as a bolometer or thermopile.

## 4 Conclusion

We have proposed an ultranarrowband, on‐chip multiwavelength sensor with parallel detection for practical and portable spectroscopic applications. As a proof of concept, we have designed and experimentally demonstrated an on‐chip quad‐wavelength membrane sensor operating in the MWIR atmospheric window region. The optical and thermal properties of the quad‐wavelength membrane sensor were numerically simulated and optimized to have narrow bandwidths (*Q*‐factor of 73) and nearly perfect absorptivities (0.99) with an efficient light–heat conversion and a direct conductive heat transfer. The fabricated sensor chips exhibited ultrasharp spectral response under normal incidence operating in the MWIR region, which appropriately proves that the conceptual design of the WAA multiwavelength IR sensor is applicable. Our work also provides a clear vision that the multiwavelength spectroscopic device can be fabricated on a single Si chip using CMOS‐compatible MEMS design and can be easily extended into multicolor IR devices. With the proposed simple design, the multicolor IR sensors presented in this work exhibit ultrasharp wavelength resolution of ≈50 nm at the normal incidence; and are expected to serve as miniature spectroscopic IR devices useful for true‐temperature pyrometry, gas imaging, position, and motion sensing with high angular resolution, materials specific imaging, as wells as environmental sensing.

## 5 Experimental Section

*Numerical Simulations*: The optical spectra (transmittance, reflectance, and absorptivity) of the 2D WAA were simulated using RCWA method (DiffractMOD, Synopsys' RSoft). For the electric field and absorption distributions, a full‐wave simulation based on FDTD method (FullWAVE, Synopsys' RSoft) was employed. For FDTD simulation, periodic boundary conditions were applied to the *x*‐ and *y*‐directions, a perfectly matched layer was applied to the *z*‐direction, and a mesh size of 2.5 nm was applied for both directions. For both RCWA and FDTD simulations, the excitation electromagnetic field propagated along the *– z*‐axis and the electric field oscillated along the *x‐*axis, the incident field amplitudes and their phases were normalized to 1. In the electromagnetic simulations, the dielectric functions of Au, Si, and SiO_2_ were taken from the literature,^51^ the dielectric function of ZnO was taken from literature,^31^ and Si_3_N_4_ was retrieved from spectroscopic ellipsometry measurement carried out using two ellipsometers (SENTECH, SE 850 DUV for UV–NIR region and SENDIRA for IR region) (see Figure S6, Supporting Information). The heat transfer simulation in the quad‐wavelength IR sensor was performed using a finite‐element method implemented in a commercial package (COMSOL Multiphysics). The absorptivity of each surface, which was obtained from the RCWA calculation, was predefined. The density, thermal conductivity, and specific heat capacity of all materials used in the heat transfer simulation are detailed in Table S1 of the Supporting Information.

*Device Fabrication*: (see also Figure 3) First, a 3 in. double side polished Si wafer was thermally oxidized at 1150 °C in dry oxidation to form an ≈100 nm thick SiO_2_ layer on both sides of the Si wafer. A 350 nm thick Si_3_N_4_ film was then deposited on both sides of the SiO_2_/Si wafer following a DC (200 W) reactive sputtering recipe with a boron‐doped Si target and a mixture of Ar/N_2_ (18/10 sccm) gases (sputter i‐Miller CFS‐4EP‐LL, Shibaura) (see Figure 3a). Subsequently, a rapid thermal annealing process in N_2_ atmosphere (heating rate of 5 °C s^−1^, keeping constant at 1000 °C for 1 min, then naturally cooling down) was applied on the sputtered Si_3_N_4_/SiO_2_/Si substrate to improve the quality (hardness) of the S_3_N_4_ film. Next, a following maskless lithography procedure to create photoresist patterns as mask for the lift‐off process of the bottom Pt film electrode was employed. First, double spin‐coated AZ‐5214E/OFPR‐800LB photoresist layers (5000 rpm spin‐coating, soft‐baking at 90 °C within 5 min for each layer) were prepared on the Si_3_N_4_/SiO_2_/Si substrate. Then, a direct laser writing exposure (405 nm laser wavelength, *µ*PG 101 Heidelberg Instruments) following a CAD drawing pattern was then applied on the double photoresist layers. After hard baking at 120 °C for 30 s and applying an image reversal exposure under UV light, the exposed double photoresist layers were developed, subsequently rinsed in distilled water and dried using a N_2_ gas blower. A 100 nm Pt film with a 10 nm adhesive Ti layer for the bottom electrode of the sensor was deposited on the Si_3_N_4_/SiO_2_/Si substrate with the patterned photoresist mask using an electron beam evaporator (UEP‐300‐2C, ULVAC). The lift‐off process was done using PG remover.

For patterning of the pyroelectric ZnO film (by sputtering) and the top Au electrode (by electron beam deposition), the same maskless lithography procedures as the above were applied. It is worth noting that here an RF (300 W) sputtering recipe with ZnO target and a mixture of Ar/O_2_ (16/04 sccm) gases for the epitaxial growth of the highly crystalline ZnO film on Pt bottom film electrode were used.^30^ After preparing the top Au electrode, a 340 nm amorphous Si (boron‐doped) film as the template layer for Au disk array was patterned on the top Au film electrode (Figure 3b). The photoresist disk arrays (AZ‐514E) designed for each quad‐wavelength sensor as an RIE mask for etching Si were patterned on the Si template using a direct laser writing lithography process (Figure 3c). Subsequently, an RIE recipe (CH_4_ plasma, Ulvac CE‐300I) was used to etch Si around the photoresist disk mask (Figure 3d). The remaining photoresist was removed by O_2_ plasma and by acetone. The Au disk array of each 2D periodic plasmonic absorber was finally formed by applying the above maskless lithography procedure with a DC sputtering of 80 nm Au film after a 5 nm adhesive Ti layer (Figure 3e). The quad‐wavelength IR sensor chips on the 3 in. wafer were then processed for the thermal isolation with membrane support. Here, AZ‐514E photoresist RIE masks (for membrane and for thermal isolation slits around each single‐wavelength sensor) of Si_3_N_4_ layer were first patterned. Then the Si_3_N_4_ mask for anisotropic wet‐etching of Si was formed using an RIE recipe (CHF_3_ plasma). After protecting the sensor chips in the top Si wafer by a polymeric protective layer (ProTEK B3‐25 on ProTEK B3 Primer), the Si substrate at the bottom of each single‐wavelength sensor was completely etched by a slow‐rate anisotropic wet‐etching using a hot KOH solution (8 mg L^−1^, 80 °C). The sensor chip wafer was then kept in PG remover for one day, and finally rinsed by acetone before separating them into 1 × 1 cm^2^ quad‐wavelength IR membrane sensor chips.

*Characterization*: SEM images of the fabricated quad‐wavelength IR membrane sensor were taken using an SEM (Hitachi SU8230) under an accelerating voltage of 5 kV. For the cross‐sectional view image, a focused ion beam miller (Hitachi FB‐2100) was used to create a rectangular through hole in a membrane sensor chip. For the spectral response measurement, a tunable IR laser system was used as a tunable excitation source. In this laser system, a one‐box ultrafast amplifier system (Solstice, Spectra‐Physics) that comprises a mode‐locked Ti:sapphire laser and a regenerative amplifier, was used to produce high‐power, ultrafast (104 fs), near infrared (800 nm) pulses (1 kHz) in a beam of exceptional quality. The spectral range of the output laser from Solstice amplifier system is then extended from UV to IR region and amplified by using a traveling‐wave optical parametric amplifier (TOPAS‐Prime, Spectra‐Physics) combined with a near‐IR, UV, vis generator (NirUVis, Light Conversion), and a non‐collinear difference frequency generator (Light Conversion). The output IR laser had following characteristics: board spectral linewidth with the *Q*‐factor of about 10–15; collimated beam with 1 mm diameter; 1 kHz repetition rate; few milliwatts average power depending on the wavelength. In the measurement, the IR sensors were directly irradiated to the 1 mm diameter laser. It should be noted that the spectral linewidth of the output IR laser is much broader compared to the absorption bandwidth of IR sensors, which caused the broadening of the spectral response of IR sensors. In the measurement, the signal from quad‐wavelength IR membrane sensor excited by the tunable IR laser was first preamplified using a voltage amplifier (SR560, Stanford Research Systems), and then gained using a lock‐in amplifier (LI5640, NF Corporation), and finally measured by a source meter (ADCMT 8252). The spectral power distribution of the output IR laser was measured using a thermal power sensor head (S401C Thorlabs) equipped with a power meter console (PM100D, Thorlabs). The spectral response of each IR sensor was calculated by dividing the spectral output voltage at the IR sensor to the measured spectral power distribution of the IR laser. In the temporal response characteristic of the fabricated IR sensor was measured using a high‐performance oscilloscope (500 MHz, Tektronix TDS 520A) combined with SR560 amplifier. The detail of the measurement setup for the spectral response and temporal response of the sensors are illustrated in Figure S7 of the Supporting Information.

## Conflict of Interest

The authors declare no conflict of interest.

## Supporting information

Supplementary

## References

[1] J. Rosenberg , R. V. Shenoi , S. Krishna , O. Painter , Opt. Express 2010, 18, 3672. 20389377 10.1364/OE.18.003672

[2] S. Kalchmair , H. Detz , G. D. Cole , A. M. Andrews , P. Klang , M. Nobile , R. Gansch , C. Ostermaier , W. Schrenk , G. Strasser , Appl. Phys. Lett. 2011, 98, 011105.

[3] S. Kalchmair , R. Gansch , S. I. Ahn , A. M. Andrews , H. Detz , T. Zederbauer , E. Mujagić , P. Reininger , G. Lasser , W. Schrenk , G. Strasser , Opt. Express 2012, 20, 5622. 22418369 10.1364/OE.20.005622

[4] X. Gan , X. Yao , R.‐J. Shiue , F. Hatami , D. Englund , Opt. Express 2015, 23, 12998. 26074552 10.1364/OE.23.012998

[5] C.‐C. Chang , Y. D. Sharma , Y.‐S. Kim , J. A. Bur , R. V. Shenoi , S. Krishna , D. Huang , S.‐Y. Lin , Nano Lett. 2010, 10, 1704. 20405905 10.1021/nl100081j

[6] W. Wu , A. Bonakdar , H. Mohseni , Appl. Phys. Lett. 2010, 96, 161107.

[7] S. J. Lee , Z. Ku , A. Barve , J. Montoya , W.‐Y. Jang , S. R. J. Brueck , M. Sundaram , A. Reisinger , S. Krishna , S. K. Noh , Nat. Commun. 2011, 2, 286. 21505442 10.1038/ncomms1283

[8] Y. L. Jing , Z. F. Li , Q. Li , X. S. Chen , P. P. Chen , H. Wang , M. Y. Li , N. Li , W. Lu , Sci. Rep. 2016, 6, 25849. 27181111 10.1038/srep25849 PMC4867604

[9] A. Wang , Y. Dan , Sci. Rep. 2018, 8, 11257. 30050145 10.1038/s41598-018-29177-0 PMC6062579

[10] B. Zheng , H. Zhao , B. Cerjan , S. Yazdi , E. Ringe , P. Nordlander , N. J. Halas , Appl. Phys. Lett. 2018, 113, 101105.

[11] N. I. Landy , S. Sajuyigbe , J. J. Mock , D. R. Smith , W. J. Padilla , Phys. Rev. Lett. 2008, 100, 207402. 18518577 10.1103/PhysRevLett.100.207402

[12] K. Aydin , V. E. Ferry , R. M. Briggs , H. A. Atwater , Nat. Commun. 2011, 2, 517. 22044996 10.1038/ncomms1528

[13] H. Wang , L. Wang , Opt. Express 2013, 21, A1078. 24514927 10.1364/OE.21.0A1078

[14] J. B. Chou , Y. X. Yeng , Y. E. Lee , A. Lenert , V. Rinnerbauer , I. Celanovic , M. Soljačić , N. X. Fang , E. N. Wang , S.‐G. Kim , Adv. Mater. 2014, 26, 8041. 25228299 10.1002/adma.201403302

[15] E. Rephaeli , S. Fan , Opt. Express 2009, 17, 15145. 19687992 10.1364/oe.17.015145

[16] L. P. Wang , Z. M. Zhang , Appl. Phys. Lett. 2012, 100, 063902. 10.1063/1.3696307 PMC3326061 22509069

[17] B. Zhao , L. Wang , Y. Shuai , Z. M. Zhang , Int. J. Heat Mass Transfer 2013, 67, 637.

[18] H. T. Miyazaki , K. Ikeda , T. Kasaya , K. Yamamoto , Y. Inoue , K. Fujimura , T. Kanakugi , M. Okada , K. Hatade , S. Kitagawa , Appl. Phys. Lett. 2008, 92, 141114.

[19] X. Liu , T. Tyler , T. Starr , A. F. Starr , N. M. Jokerst , W. J. Padilla , Phys. Rev. Lett. 2011, 107, 045901. 21867022 10.1103/PhysRevLett.107.045901

[20] T. D. Dao , K. Chen , S. Ishii , A. Ohi , T. Nabatame , M. Kitajima , T. Nagao , ACS Photonics 2015, 2, 964.

[21] T. Yokoyama , T. D. Dao , K. Chen , S. Ishii , R. P. Sugavaneshwar , M. Kitajima , T. Nagao , Adv. Opt. Mater. 2016, 4, 1987.

[22] E. Rephaeli , A. Raman , S. Fan , Nano Lett. 2013, 13, 1457. 23461597 10.1021/nl4004283

[23] A. P. Raman , M. A. Anoma , L. Zhu , E. Rephaeli , S. Fan , Nature 2014, 515, 540. 25428501 10.1038/nature13883

[24] T. Liu , J. Takahara , Opt. Express 2017, 25, A612. 28788827 10.1364/OE.25.00A612

[25] H. T. Miyazaki , T. Kasaya , M. Iwanaga , B. Choi , Y. Sugimoto , K. Sakoda , Appl. Phys. Lett. 2014, 105, 121107.

[26] A. Lochbaum , Y. Fedoryshyn , A. Dorodnyy , U. Koch , C. Hafner , J. Leuthold , ACS Photonics 2017, 4, 1371.

[27] K. Chen , R. Adato , H. Altug , ACS Nano 2012, 6, 7998. 22920565 10.1021/nn3026468

[28] K. Chen , T. D. Dao , S. Ishii , M. Aono , T. Nagao , Adv. Funct. Mater. 2015, 25, 6637.

[29] F. B. P. Niesler , J. K. Gansel , S. Fischbach , M. Wegener , Appl. Phys. Lett. 2012, 100, 203508.

[30] S. Ogawa , K. Okada , N. Fukushima , M. Kimata , Appl. Phys. Lett. 2012, 100, 021111.

[31] T. D. Dao , S. Ishii , T. Yokoyama , T. Sawada , R. P. Sugavaneshwar , K. Chen , Y. Wada , T. Nabatame , T. Nagao , ACS Photonics 2016, 3, 1271.

[32] A. Ahmed , H. Kim , J. Kim , K. Hwang , S. Kim , Sensors 2017, 17, 908.

[33] J. Y. Suen , K. Fan , J. Montoya , C. Bingham , V. Stenger , S. Sriram , W. J. Padilla , Optica 2017, 4, 276.

[34] S. Ishii , K. Uchida , T. D. Dao , Y. Wada , E. Saitoh , T. Nagao , APL Photonics 2017, 2, 106103.

[35] J. H. Poynting , Philos. Trans. R. Soc. London 1884, 175, 343.

[36] D. J. Griffiths , Introduction to Electrodynamics, Prentice Hall, Englewood Cliffs, NJ, USA 1999.

[37] R. Loudon , J. Phys. A: Gen. Phys. 1970, 3, 233.

[38] R. Ruppin , Phys. Lett. A 2002, 299, 309.

[39] J. Hao , L. Zhou , M. Qiu , Phys. Rev. B 2011, 83, 165107.

[40] R. W. Wood , Proc. Phys. Soc. London 1902, 18, 269.

[41] J. Strong , Phys. Rev. 1936, 49, 291.

[42] U. Fano , J. Opt. Soc. Am. 1941, 31, 213.

[43] A. Hessel , A. A. Oliner , Appl. Opt. 1965, 4, 1275.

[44] R. H. Ritchie , E. T. Arakawa , J. J. Cowan , R. N. Hamm , Phys. Rev. Lett. 1968, 21, 1530.

[45] E. Mirica , G. Kowach , P. Evans , H. Du , Cryst. Growth Des. 2004, 4, 147.

[46] J. L. Gardner , T. P. Jones , J. Phys. E 1980, 13, 306.

[47] N. Narang , T. Bourlai , Image Vision Comput. 2015, 33, 26.

[48] B. N. Duncan , A. I. Prados , L. N. Lamsal , Y. Liu , D. G. Streets , P. Gupta , E. Hilsenrath , R. A. Kahn , J. E. Nielsen , A. J. Beyersdorf , S. P. Burton , A. M. Fiore , J. Fishman , D. K. Henze , C. A. Hostetler , N. A. Krotkov , P. Lee , M. Lin , S. Pawson , G. Pfister , K. E. Pickering , R. B. Pierce , Y. Yoshida , L. D. Ziemba , Atmos. Environ. 2014, 94, 647.

[49] P. Gaudio , M. Gelfusa , A. Malizia , S. Parracino , M. Richetta , L. De Leo , C. Perrimezzi , C. Bellecci , J. Phys.: Conf. Ser. 2015, 658, 012004.

[50] S. Fanchenko , A. Baranov , A. Savkin , A. Somov , L. Calliari , 2016 IEEE Int. Smart Cities Conf. ISC2, IEEE, Piscataway, NJ, USA 2016, 10.1109/ISC2.2016.7580867.

[51] E. D. Palik , Handbook of Optical Constants of Solids, Academic Press, New York 1998.

