# Supplementary material for: An On‐Chip Quad‐Wavelength Pyroelectric Sensor for Spectroscopic Infrared Sensing
Source: Adv Sci (Weinh). 2019 Aug 26;6(20):1900579. doi: 10.1002/advs.201900579 (PMC6794626; doi:10.1002/advs.201900579)
Supplement: Supplementary file 1 — Supplementary [file ADVS-6-1900579-s001.pdf]

Copyright WILEY-VCH Verlag GmbH & Co. KGaA, 69469 Weinheim, Germany, 2019.

ADVANCED SCIENCE
Open Access

Supporting Information

for *Adv. Sci.,* DOI: 10.1002/advs.201900579

An On-Chip Quad-Wavelength Pyroelectric Sensor for Spectroscopic Infrared Sensing

Thang Duy Dao, Satoshi Ishii, Anh Tung Doan, Yoshiki Wada, Akihiko Ohi, Toshihide Nabatame, and Tadaaki Nagao**

Copyright WILEY-VCH Verlag GmbH & Co. KGaA, 69469 Weinheim, Germany, 2019.

Supporting Information

On-Chip Quad-Wavelength Pyroelectric Sensor for Spectroscopic Infrared Sensing

Thang Duy Dao, Satoshi Ishii, Anh Tung Doan, Yoshiki Wada, Akihiko Ohi, Toshihide Nabatame, Tadaaki Nagao**

Dr. T. D. Dao, Dr. S. Ishii, A. T. Doan, Prof. T. Nagao
International Center for Materials Nanoarchitectonics (MANA), National Institute for Materials Science (NIMS), 1-1 Namiki, Tsukuba, Ibaraki 305-0044, Japan.
E-mail: Dao.duythang@nims.go.jp; Nagao.Tadaaki@nims.go.jp

Dr. Y. Wada
Research Center for Functional Materials, National Institute for Materials Science (NIMS), 1-1 Namiki, Tsukuba, Ibaraki 305-0044, Japan.

Dr. A. Ohi, Dr. T. Nabatame
Nanotechnology Innovation Station, National Institute for Materials Science (NIMS), 1-1 Namiki, Tsukuba, Ibaraki 305-0044, Japan.

A. T. Doan, Prof. T. Nagao
Department of Condensed Matter Physics, Graduate School of Science, Hokkaido University, Kita 8, Nishi 5, Kita-ku, Sapporo 060-0810, Japan

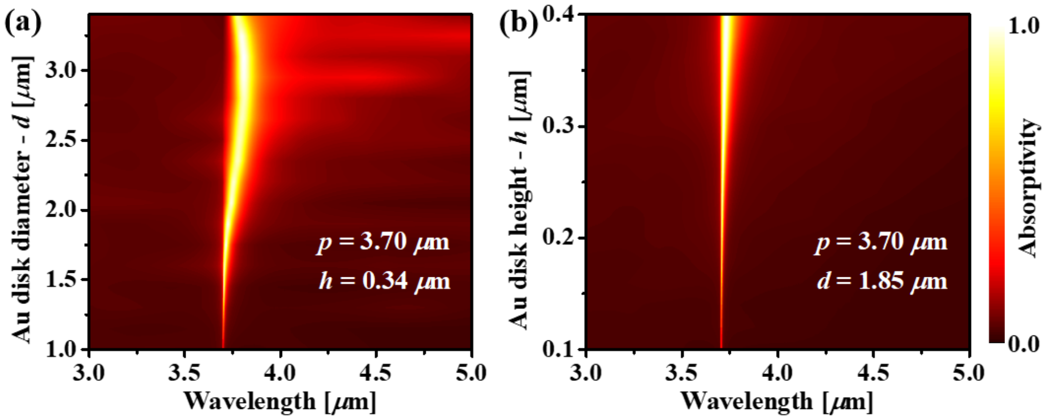

Figure S1. Simulated dependences of (a) disk diameter and (b) disk height on the absorptivity of a 3.7 μm single-wavelength membrane pyroelectric sensor.

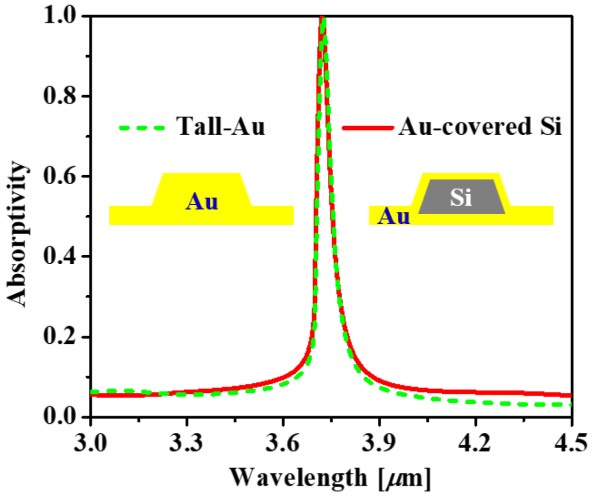

Figure S2. Simulated absorptivity spectra of tall Au disk (dashed green curve) and Au-covered Si disk (solid red curve) Wood's anomaly absorbers having identical parameters (periodicity of 3.7 μm, disk diameter of 1.85 μm and height of 0.34 μm). Both two Wood's anomaly absorber configirations exhibit the same performance with a nearly perfect absorptivity (0.99) resonance at 3.722 μm and a narrow bandwidth of 51 nm (Q-factor of 73).

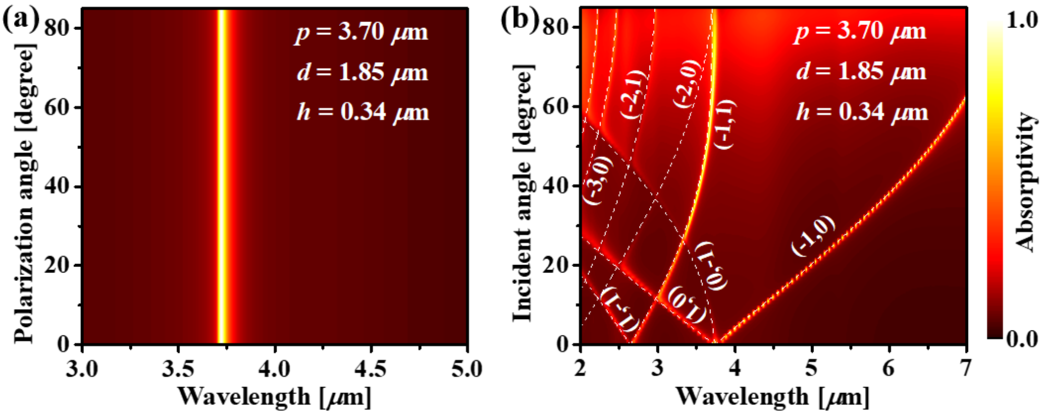

Figure S3. Simulated (a) polarization independence and (b) angle dependence on the absorptivity of a 3.7 μm single-wavelength membrane pyroelectric sensor. White-dashed curves indicate SPPs dispersion relation in the 2D periodic plasmonic square lattice.

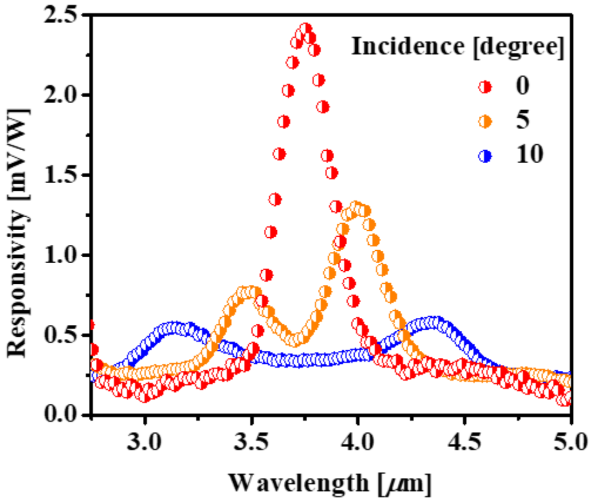

Figure S4. Measured angle-dependent spectral response of the 3.722 μm resonant sensor chip.

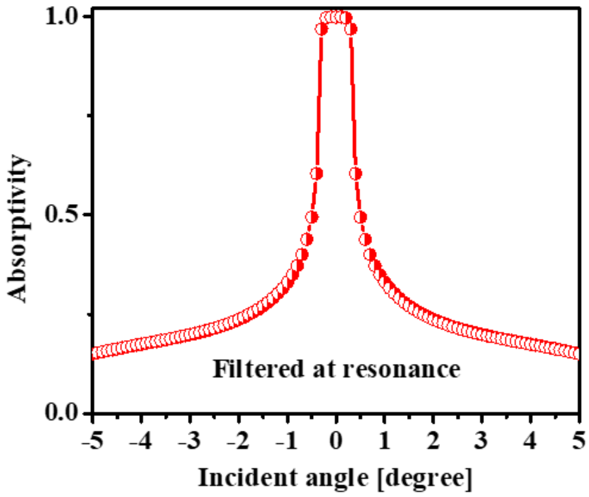

Figure S5. Simulated angle-dependent absorptivity of the sensor chip filtered at the resonance.

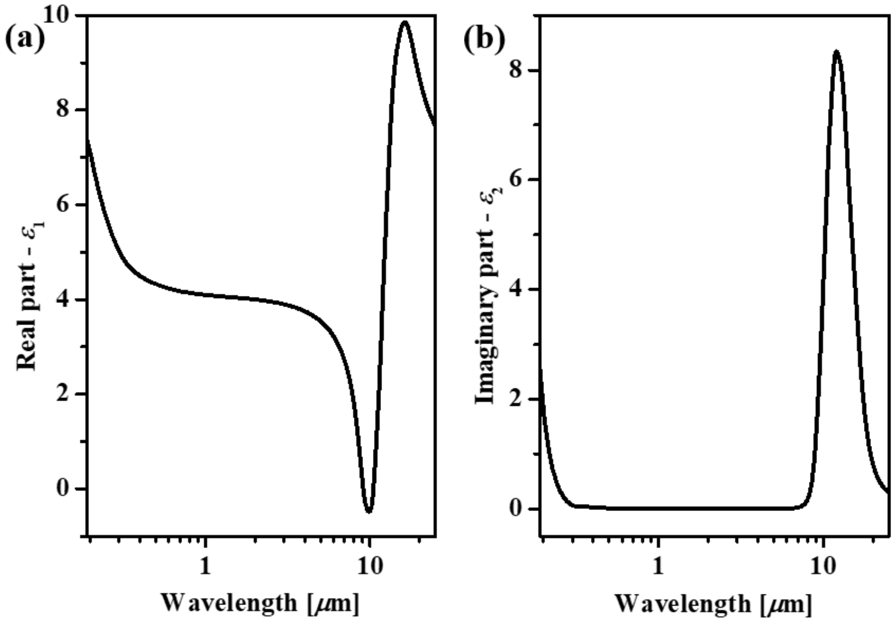

Figure S6. (a) Real and (b) imaginary parts of the retrieved complex dielectric function of the sputtered Si_3N_4 film.

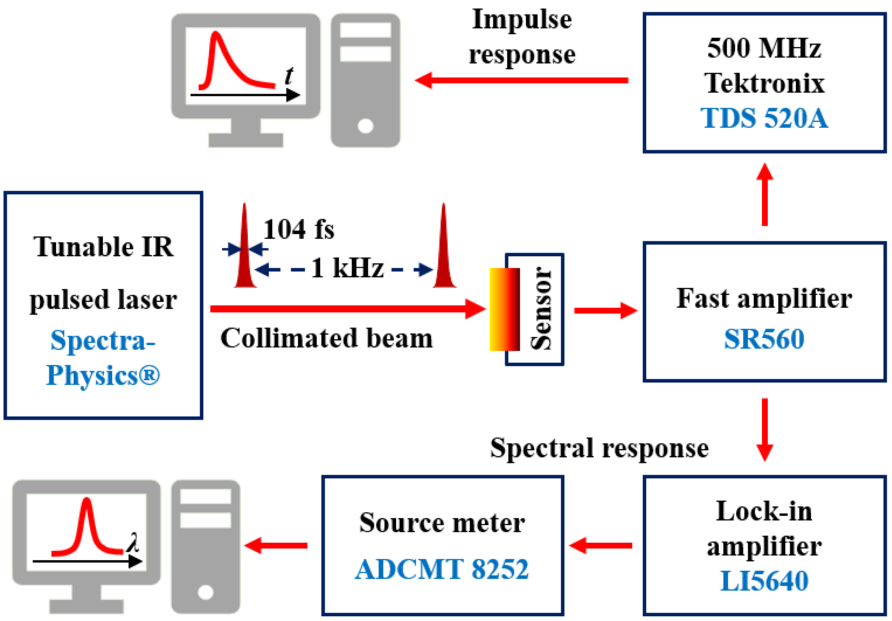

Figure S7. Schematic illustration of the measurement setup for the spectral response and temporal response of the on-chip quad-wavelength IR sensor.

Table S1. The density, thermal conductivity and specific heat capacity of all materials used in the heat transfer simulations.

Materials	Density [g·cm^{-3}]	Thermal conductivity [W·m^{-1}·K^{-1}]	Specific heat capacity [J·g^{-1}·K^{-1}]
Au	19.30[1]	293[1]	0.126[1]
Si	2.34[1]	149[2]	0.712[2]
Pt	21.45[1]	71.6[3]	0.126[3]
ZnO	5.675[4]	54[5]	0.494[4]
Si_3N_4	3.17[4]	43[4]	1.1[4]
SiO_2	2.196[4]	1.4[4]	0.73[4]

References:

[1] E. A. (Eric A. Brandes, G. B. Brook, *Smithells Metals Reference Book.*, Butterworth-Heinemann, **1999**.

[2] S. M. Sze, K. K. Ng, *Physics of Semiconductor Devices*, Wiley-Interscience, **2007**.

[3] F. Cverna, ASM International. Materials Properties Database Committee., *ASM Ready Reference. Thermal Properties of Metals*, ASM International, **2002**.

[4] W. M. Haynes, *CRC Handbook of Chemistry and Physics : A Ready-Reference Book of Chemical and Physical Data*, CRC Press, **2011**.

[5] J. Alvarez-Quintana, E. Martínez, E. Pérez-Tijerina, S. A. Pérez-García, J. Rodríguez-Viejo, *J. Appl. Phys.* **2010**, *107*, 063713.
